# Biomarkers and indoor air quality: A translational research review

**DOI:** 10.1017/cts.2020.532

**Published:** 2020-09-04

**Authors:** Araliya M. Senerat, Sheila M. Manemann, Nicholas S. Clements, Robert D. Brook, Leslie C. Hassett, Véronique L. Roger

**Affiliations:** 1 Well Living Lab, Inc., Mayo Clinic, Rochester, MN 55902, USA; 2 Department of Health Sciences Research, Mayo Clinic, Rochester, MN 55905, USA; 3 Department of Internal Medicine, University of Michigan Medical School, Ann Arbor, MI 48109, USA; 4 Library Public Services, Mayo Clinic, Rochester, MN 55905, USA; 5 Department of Cardiovascular Diseases, Mayo Clinic, Rochester, MN 55905, USA

**Keywords:** Indoor air quality, biomarkers, air pollution, ambient air, inflammation, oxidative stress

## Abstract

**Introduction:**

Air pollution is linked to mortality and morbidity. Since humans spend nearly all their time indoors, improving indoor air quality (IAQ) is a compelling approach to mitigate air pollutant exposure. To assess interventions, relying on clinical outcomes may require prolonged follow-up, which hinders feasibility. Thus, identifying biomarkers that respond to changes in IAQ may be useful to assess the effectiveness of interventions.

**Methods:**

We conducted a narrative review by searching several databases to identify studies published over the last decade that measured the response of blood, urine, and/or salivary biomarkers to variations (natural and intervention-induced) of changes in indoor air pollutant exposure.

**Results:**

Numerous studies reported on associations between IAQ exposures and biomarkers with heterogeneity across study designs and methods. This review summarizes the responses of 113 biomarkers described in 30 articles. The biomarkers which most frequently responded to variations in indoor air pollutant exposures were high sensitivity C-reactive protein (hsCRP), von Willebrand Factor (vWF), 8-hydroxy-2′-deoxyguanosine (8-OHdG), and 1-hydroxypyrene (1-OHP).

**Conclusions:**

This review will guide the selection of biomarkers for translational studies evaluating the impact of indoor air pollutants on human health.

## Introduction

Air quality impacts human health [1,2]; airborne contaminants include fine particulate matter (PM_2.5_, airborne particles with diameters less than 2.5 µm), ozone (O_3_), volatile organic compounds (VOCs), and biological particles (e.g., allergens and pathogens). Since individuals spend about 90% of their time indoors, indoor air quality (IAQ) is a key driver of the effect of air quality on human health [3,4]. In particular, IAQ is linked to cardiovascular [5] and respiratory morbidity [6,7] and mortality [8–11]. Modeling data estimated that indoor exposure to PM_2.5_ accounts for the vast majority of the mortality burden being attributed to total exposure to PM_2.5_ [10]. To evaluate the effectiveness of interventions to improve IAQ, one must study relevant outcomes. Cardiovascular and respiratory events can take a long time to accrue and be challenging to study in a randomized design. Thus, intermediate endpoints that respond to natural or intervention-induced changes in IAQ are critical to research in this field. The American Heart Association Scientific Statement on air pollution and cardiovascular disease underscored the need to “better describe the physiological relevance in humans and the fundamental details of the mechanisms” [2].

The goal of the present review is to address this stated need and summarize current knowledge on biomarkers associated with IAQ exposure in order to guide the design of translational research studies on indoor air quality.

## Methods

### Data Sources and Search Strategies

A comprehensive search was conducted from January 1, 2000 to September 17, 2019 to identify studies that reported on blood, urine, and salivary biomarkers relevant to indoor air pollution exposure and toxicology. Breath biomarkers were beyond our intended scope and are not addressed herein. The search strategy was designed and conducted by an experienced librarian (L.C.H.) with input from investigators (A.M.S. and S.M.M.) and was performed in Ovid Medline, Ovid Embase, Ovid Cochrane Central Register of Controlled Trials, Ovid Cochrane Database of Systematic Reviews, and Scopus. Controlled vocabulary supplemented with keywords was used, the search was limited to the English language, and animal studies were excluded. The full search strategy is included in the online supplemental Appendix 1.

### Study Selection

**Fig. 1.**
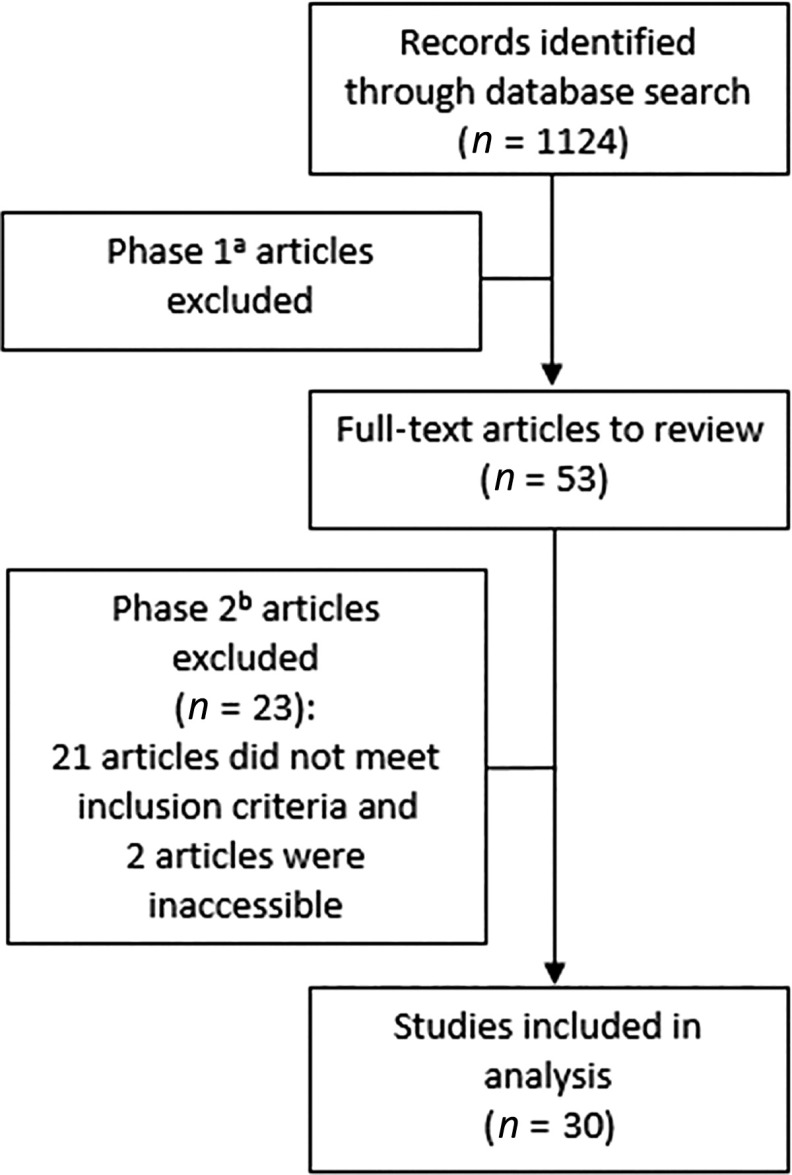
Flow diagram illustrating the methods applied to the review. ^a^Phase 1 of the review involved reviewing the title and abstract, and excluded studies that involved only children, factory workers, or pregnant women, involved biomass, coal, or open wood-burning studies; focused only on tobacco, lead, or dust exposures. ^b^Phase 2 involved reviewing the full-text papers and used the same exclusion criteria as Phase 1.

A total of 1124 papers were identified. Phase 1 involved 2 investigators (A.M.S. and S.M.M.) reviewing all titles and abstracts. We included all English language original research studies with at least 10 adult participants published over the last decade between January 1, 2010 and September 17, 2019. Only studies that measured biomarkers in blood, urine, or saliva and focused on indoor exposures were included. We excluded studies that involved only children, factory workers, or pregnant women, involved biomass, coal, or open wood-burning studies; focused only on tobacco, lead, or dust exposures. Studies with industrial settings were excluded because indoor pollutants that may be encountered in industrial settings are not representative of indoor exposures in most buildings, including homes, offices, schools, and healthcare settings. In doing so, we selected 53 full-text papers for analysis. Phase 2 involved 2 investigators (A.M.S. and S.M.M.) reviewing the full-text papers. Data reviewed included the type of biomarkers and specimen type (blood, urine, and saliva), country, setting (home, office, etc.), seasons, frequency of data collection, study length, intervention type, population type and size, air pollutant levels and types, and a summary of methods and results. Among these, 23 papers were excluded: 21 did not meet the inclusion criteria (1 article had no mention of biomarkers, 7 collected air exposure measurements off-site, 8 had no mention of IAQ exposures, 1 focused on factory workers, 3 used coal/biomass/open wood burning, 1 included participants with a disease), and 2 were inaccessible. Thirty articles were retained for the final analyses (Fig. 1).

## Results

The thirty studies included sample sizes ranging from 20 to 200 participants (Table 1). Participants’ age ranged from 15 to 90, and originated from 11 countries (5 in the USA, 7 in China, 5 in Taiwan, 1 in South Korea, 8 in Europe, 1 in Iran, 1 in Senegal, and 2 in India). Most studies (18 out of 30) consisted of non-randomized comparisons across different settings with a few observational monitoring. Nineteen of the studies were observational and/or cross-sectional studies, while the remaining 11 studies were interventional and/or crossover trials. More details regarding study design can be found in Table 1. Almost half of the studies (*n* = 12) measured biomarkers at only one time point. Out of 30 studies, 3 provided an estimate of their statistical power to observe a change.

**Table 1.** Summary of IAR studies measuring physiological biomarkers and organic compounds in humans

Citation	Location	Setting	Design	N^a^	Study duration and collection time points	Biomarkers measured
Physiological biomarkers						
Brugge (2017) [22]	USA	Home	Double-blind, randomized crossover trial comparing HEPA versus sham filtration in the same group of participants	23	Blood collected 3x over 6 weeks (at baseline, week 3, and post-intervention) and air exposures measured continuously	Blood: TNF-RII, IL-6, hsCRP
Chen (2015) [14]	China	Dorms	Randomized double-blind crossover trial comparing air filtration purifier versus sham filtration among two independent groups	35	Blood collected 3x (at baseline, after 2 days of air filtration purifier, and after 2 days of sham filtration) and air exposures measured on an hourly basis for 4 days over a 2-week period	Blood: CRP, fibrinogen, P-selectin, MCP-1^*^, IL-1β^*^, IL-6, TNF-α, myeloperoxidase^*^, sCD40L^*^, PAI-1, t-PA^*^, D-Dimer, endothelin-1, angiotensin-converting enzyme
Wang (2011) [34]	China	Kitchen	Cross-sectional comparison of occupational exposures between two independent groups of kitchen versus non-kitchen workers	110	1 day, with blood collected 1x and air exposures measured twice during lunch and dinner hours	Blood: lymphocytic BNMNs, Comet assay variables (tail length^*^ and tail DNA%), SOD, and MDA^*^ Urine: 1-OHP, 8-oxodG
Chuang (2017) [19]	Taiwan	Home	Randomized crossover intervention comparing air filtration intervention versus control (false air conditioner filter) in the same group of participants	200	Twelve visits at 2-month intervals over 2 years, with blood and air exposures collected at each visit	Blood: hsCRP^*^, 8-OHdG^*^, and fibrinogen
Cui (2018) [23]	China	Home	Double-blind randomized crossover study comparing HEPA versus Sham filtration among the same group of participants	70	4 days with blood collected before and after filtration systems and air exposures monitored before, during, and after filtration systems	Blood: IL-6^*^, vWF^*^, and sCD62P Urine: MDA
Day (2018) [12]	China	Office and dorms	Intervention comparing three ventilation systems (F8-ESP-HEPA, F8 only, F8 + HEPA) across two independent groups	89	5 weeks with four biomarker collections (pre-intervention, 2x during intervention, and post-intervention) and air exposures measured continuously	Blood: CRP, 8-OHdG, sCD62P^*^, VWF^*^ Urine: MDA
Hassanvand (2017) [20]	Tehran, Iran	Retirement home and dorm	Cross-sectional study monitoring of pollutants across two independent groups	84	1 year with six blood collections every 2 months and 24-hour exposure sampling every 2 months	Blood: WBC^*^, hsCRP^*^, sTNF-RII^*^, IL-6^*^, vWF^*^
Jung (2014) [24]	Taiwan	Office	Cross-sectional study monitoring pollutants over 1-day physiological measurements collected at end of workday across the same group of participants	115	1 day with biomarkers collected at the end of the workday and air exposures monitored during office hours	Urine: epinephrine^*^, norepinephrine^*^, cortisol^*^, creatinine, 8-OHdG^*^ Saliva: IL-6 and TNF-a
Matthews (2010) [38]	UK	Home	Cross-sectional comparison of heating types (piped gas, coal, electricity, liquid propane gas) across independent groups of participants	80	Air exposures measured every 5 min over 7 days and blood collected 2x: once during the week and post-6 months to account for seasonal effects	Blood: cGMP
Ndong Ba (2019) [25]	Senegal	Home	Cross-sectional study monitoring pollutants over 18 days compared across jobs and rural residence among independent groups of participants	116	Air exposures measured during working hours over 2.5 weeks and urine collected at the end of each day	Urine: S-PMA^*^, t,t-MA, 1- OHP^*^, 8-OHdG^*^, TNF-a, IL-1b, IL-6 and IL-8
Olsen (2014) [18]	Denmark	Home	Cross-sectional study monitoring pollutants over 2 days using personal and stationary monitoring across independent participants	81	Air exposures monitored over 2 days and blood collected after the monitoring.	Blood: CRP, leukocytes^*^
Pan (2011) [33]	Taiwan	Restaurant	Intervention comparing exposures before and after installation of embracing air curtain device in the same group of participants	45	Air monitoring and urine collected during the weekend before and 4 weeks after installation	Urine: 8-OHdG^*^, MDA^*^
Shao (2017) [13]	China	Home	Randomized crossover intervention comparing HEPA versus Sham filtration in the same group of participants	35	2 weeks of HEPA and 2 weeks of sham, with air exposures measured continuously and blood collected at baseline, end of HEPA, and end of sham	Blood: IL-6, IL-8^*^, CRP, Fibrinogen, 8-OHdG
Karottki (2013) [15]	Denmark	Home	Randomized, double-blind crossover intervention comparing recirculated particle-filtered versus sham-filtered indoor air in the same group of participants	48	Air exposures continuously measured over 4 weeks (2 weeks of each intervention) and blood collected at baseline and at days 2, 7, and 14 of each exposure scenario.	Blood: CRP, leukocytes, CC16, SPD, CD11b, CD31, CD49, CD62L^*^, hemoglobin
Karottki (2014) [17]	Denmark	Home	Cross-sectional study monitoring pollutants across independent participants	78	Air exposures continuously measured over 2 days and blood measured immediately after	Blood: CRP^*^, HbA1c^*^, Leukocytes^*^, lymphocytes^*^, monocytes^*^, neutrophils, eosinophils^*^, CD31, CD62L, CD11b^*^, and CD49
Karottki (2015) [16]	Denmark	Home	Intervention comparing air filtration versus sham filtration in the same group of participants	48	Seven home visits occurred over a 4-week period across 1.5 years, with air exposures measured on a weekly basis and blood collected during each home visit	Blood: Blood leukocyte counts, monocyte expression of adhesion molecules (CD31, CD62, CD11b^*^, CD49), CRP, CC16, SPD, total cholesterol, HDL, LDL, and triglycerides
Lin (2013) [21]	Taiwan	Home	Intervention comparing: (windows open, closed, closed with AC on) in the same group of participants	300	Six home visits over 6 weeks, collecting 24 hour continuous air exposures and blood during each home visit	Blood: hsCRP^*^, 8-OHdG^*^, and fibrinogen^*^
Organic compounds						
Fitzgerald (2011) [43]	USA	Home	Cross-sectional comparison of independent residents with high versus low levels of PCB exposure	253	Air samples were collected over 1 day and blood were collected after	Blood: PCB congeners 28^*^, 74, 99, 105^*^, 118, 138, 153/132, 170, 180, 183, 187, 194, and sum PCBs
Cequier (2015) [46]	Norway	Home	Cross-sectional study monitoring of pollutants one time in living rooms of independent mother–child cohorts	102	Air samples were collected over 1 day and blood were collected after	Blood: HBB, DDC-DBF, anti-DDC-CO, syn-DDC-CO, BTBPE, DDC-Ant, DBHCTD, DBDPE, sum DDC-CO
Bennett (2015) [47]	USA	Home	Longitudinal observational study monitoring pollutants twice a year apart throughout the same group of participants	139	Air and blood collected at baseline and 1 year later	Blood: pentaBDE congeners, including BDE47^*^, 99^*^, 100, 153, 154
Ke (2016) [35]	China	Kitchen	Comparative observational study comparing exposures in independent groups of staff according to frying oil exposure	236	Air samples collected over 12 hours during 2 days and urine collected pre- and post-shifts	Urine: 1-OHP^*^, 8-OHdG^*^, MDA
Kraft (2018) [62]	Germany	Office	Cross-sectional study comparing different PCBs among independent participants	35	Blood collected 1x and air sampling was measured during working hours	Blood: PCB 4^*^, 22^*^, 26^*^, 28^*^, 31^*^
Kwon (2018) [30]	South Korea	Hospital	Intervention comparing exposures when moved from old to new hospital building in the same group of participants	34	Air exposures measured in both buildings just before moving and urine collected 7 days pre-move and 7 days post-move	Urine: tt-MA^*^; HA; MA; PGA; MHA^*^; MDA, 8-OHdG, uLTE4^*^
Lai (2013) [32]	Taiwan	Office and kitchen	Longitudinal observational study comparing exposures in two independent groups of cooks versus office-based soldiers	98	Urine collected pre- and post-shifts and air sampling collected over 5 days	Urine: 1-OHP^*^ and 8-OHdG^*^
Li (2019) [50]	China	University dorms, offices, labs)	Observational pilot study monitoring pollutants across the same group of participants	20	Air samples were collected on 7 consecutive days in four seasons of 1 year and urine collected 1x each season	Urine: urinary OH-PAHs (1-OHPyr^*^, 1-OHNap^*^, 2-OHNap^*^, 2-OHFlu, 9-OHFlu^*^, 1-OHPhe, 2-OHPhe, 3-OHPhe, 4-OHPhe^*^, 9-OHPhe^*^)
Meyer (2013) [44]	Denmark	Home	Stratified cross-sectional study comparing two independent groups of participants living in non-contaminated PCB flats versus contaminated PCB flats	273	Air samples were collected over 2 months and blood collected 1x at the beginning of the study	Blood: 27 PCB congeners in plasma (congener 28^*^, 52^*^, 66^*^, 74^*^, 77, 81, 99^*^, 101^*^, 105, 114*, 118*, 123*, 126, 138, 153, 156, 157, 167, 169, 170, 178, 180, 182, 183, 187, 189, and 190)
Fraser (2012) [42]	USA	Office	Cross-sectional comparison of exposures in independent groups of participants working in new building, building renovated 1 year prior and buildings with no recent renovation	31	Air samples were collected over 4 days and blood was collected at the end of the study	Blood: PFCs (PFOA^*^, PFNA^*^, PFDA, PFHxS, PFOS)
Fraser (2013) [41]	USA	Office, homes, and vehicles	Cross-sectional comparison of exposures in independent groups of participants working in new building, building renovated 1 year prior and buildings with no recent renovation	31	Air samples were collected over 4 days and blood was collected at the end of the study	Blood: PFCs (PFOA^*^, PFNA, PFDA, PFHxS, PFOS)
Singh, Chandrasek-haran (2016) [49]	India	Kitchen	Cross-sectional comparison of exposures in independent groups of kitchen workers versus controls	188	Air samples were collected over 1 day and urine was collected 1x	Urine: PAH metabolites (1-NAP^*^, 9-PHN^*^, 1-OHP^*^, 3-HF^*^, 2-OHFlu^*^, 9-OHFlu^*^)
Singh, Kamal (2016) [48]	India	Kitchen	Cross-sectional comparison of exposures in independent groups of kitchen workers versus controls	188	Air samples were collected over 1 day and urine was collected 1x	Urine: PAH metabolites (1-NAP^*^, 9-PHN^*^, 1-OHP^*^, 3-HF^*^, 2-OHFlu^*^, 9-OHFlu^*^)

^a^N indicates the sample size of each study.

* Denotes significant changes seen in biomarkers.

**Fig. 2.**
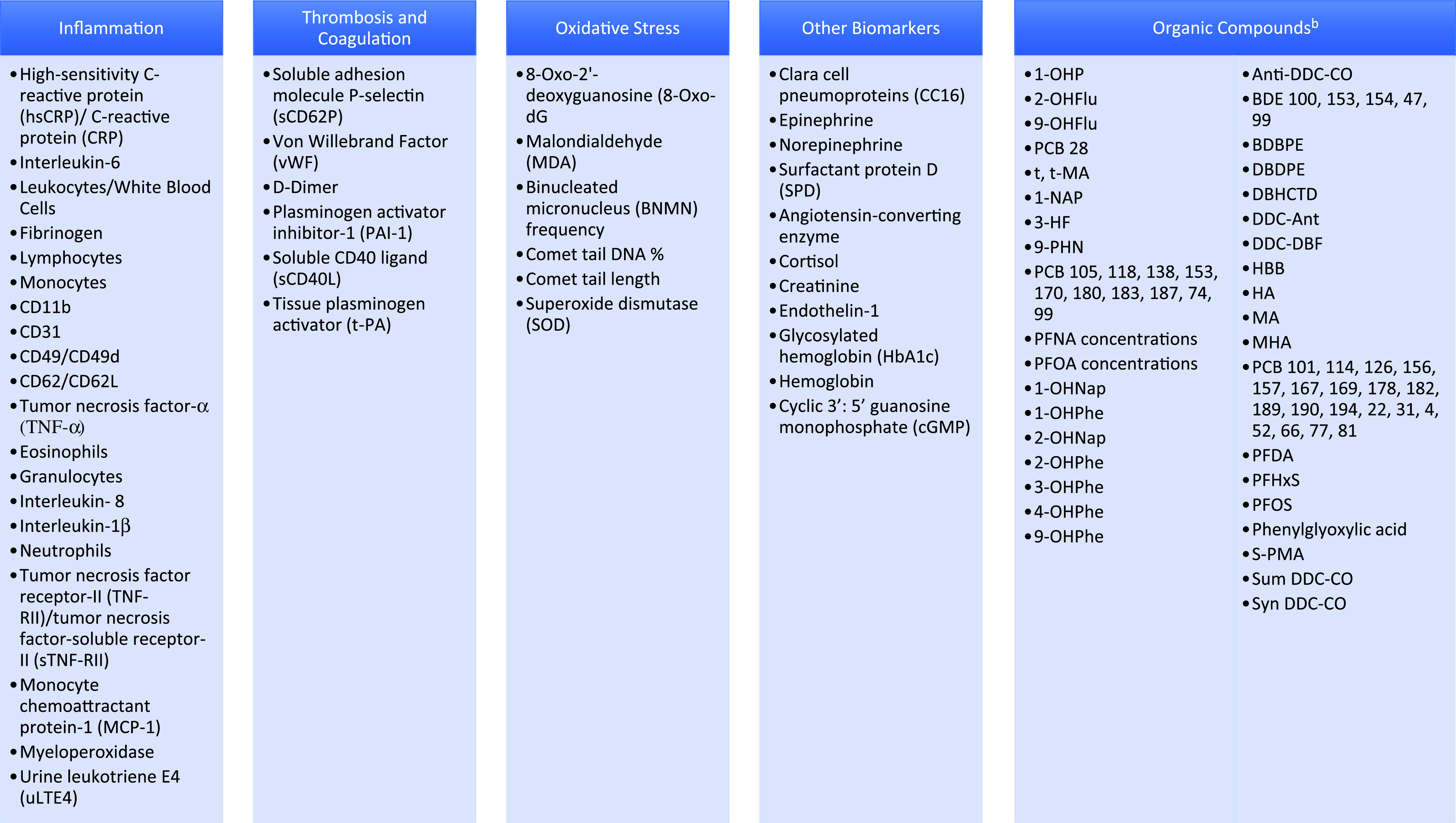
Blood, urine, and saliva biomarkers identified in IAQ papers.^aa^Biomarkers are listed in order of most frequently reported variations in response to IAQ exposures. ^b^Abbreviations can be found in Fig. 3.

One-hundred and thirteen biomarkers were identified within the 30 articles: 83 blood biomarkers, 24 urine biomarkers, 4 found in blood or urine, and 2 were found in blood, urine, or saliva. Biomarkers are presented according to the biological pathways studied, which are centered chiefly around inflammation, coagulation, and oxidative stress (Table 1). Organic compounds are considered separately. Figure 2 shows the biomarkers listed in order of most frequently reported variations in response to IAQ exposures.

## Inflammation

*C-reactive protein (CRP)* is the most frequently reported biomarker. Among 11 studies, 7 measured CRP and 4 hsCRP. Five studies evaluated a filtration system in home and/or office settings [12–16] while the remaining two monitored pollutants over time in home and/or office settings [17,18]. Only one study detected an association between PM_2.5_ and CRP [12–15,17]. Exposures evaluated included: mostly PM_2.5_ mass concentrations and/or total VOCs; [12–21] particle number concentrations (PNCs), black carbon and O_3_ [12,13,16–18]. Among the four hsCRP studies, two studies evaluated a filtration system [19,22], one evaluated an air conditioning (AC) unit [21], and one monitored pollutants over time [20]. Most studies detected significant relationships between PM_2.5_ mass concentrations and hsCRP in a home setting. Levels of hsCRP also increased with increased total VOC exposures in a home setting [19,21] and PM_10_, PM_10–2.5_, and PM_1–2.5_ mass concentrations in a retirement home setting [20]. There were no associations between hsCRP and CO_2_ or CO [21].

*Interleukins* were measured in numerous studies, with IL-6 being the most reported. Of seven papers, four compared a sham filtration system with an active filtration system [13,14,22,23] and three monitored pollutants over 1 day [24] or over time [20,25]. With regards to exposures, five papers measured PM_2.5_ mass concentrations [13,14,20,23,24]. Additional exposures were measured: CO, CO_2_, and TVOCs [24]; PM_10_, PM_10–2.5_, PM_1–2.5_, and PM_1_ [20]; black carbon; [13] O_3_, NO_2_, and PNC; [23] PNC; [22] and VOCs and PM_10_ [25]. Only two papers detected an association between IL-6 and PM_10_, PM_10–2.5_, and PM_1–2.5_ [20]. A decrease in IL-6 was reported 1 day after the installation of a high-efficiency particulate air (HEPA) filtration system [23]. The evidence of an association between air pollution and IL-8 and IL-1*β* is scarce.

Four studies measured blood *fibrinogen* in home or dormitories: three compared a sham and active filtration system [13,14,19], and one compared air quality when windows were open, closed, and when AC was on [21]. All four studies measured indoor PM_2.5_. Additional exposures measured included black carbon; [13] TVOCs; [19] and PM_10_, TVOCs, CO_2_, and CO [21]. Only one [21] study detected an association between fibrinogen and PM_2.5_ and TVOCs. Fibrinogen approached statistical significance in one study where participants were exposed to relatively higher PM_2.5_ and TVOCs [19]. The value of fibrinogen to study IAQ pollution appears marginal, calling for further research.

*Tumor Necrosis Factor-α* (TNF-α) was measured in three studies: one study compared true air filtration with a sham system; [14] two studies monitored pollutants over time [24,25]. The following exposures were measured: PM_2.5_ [14,24], VOCs [24,25], PM_10_ [25], CO [24], and CO_2_ [24]. No significant association was found between TNF-*α* and any indoor air pollutants measured. Of note, a prior review of air pollution biomarkers that combined indoor and outdoor air studies indicated that TNF-*α* was a reliable indicator of inflammation [26]. This discrepancy underscores the importance of stratifying the review of the literature by location as performed herein.

*Tumor necrosis factor-receptor II (TNF-RII) and tumor necrosis factor-soluble receptor-II (sTNF-RII)* were measured in two studies: one study compared sham filtration and HEPA filtration systems [22] and another study monitored pollutants over time [20]. No association was detected between PNC and TNF-RII [22]. However, an association was detected between sTNF-RII and PM_2.5_, PM_1_, and PM_1–2.5_ [20]. This is another domain where more research is clearly needed.

*Leukocytes* including lymphocytes, monocytes, and granulocytes (neutrophils and eosinophils) were measured in five studies; lymphocytes and monocytes were measured in four; granulocytes, neutrophils, and eosinophils were measured in two. Two studies compared sham and active filtration systems [15,16], while three monitored pollutants over time [17,18,20]. One report pertained to PM_2.5_ [15], three measured indoor air exposures to PM_2.5_ and PNC [16–18], and one measured PM_10_, PM_10–2.5_, PM_2.5_, PM_1–2.5_, and PM_1_ [20]. Significant associations were seen for the following: leukocyte counts and PNC [17,18] or PM_10_, PM_10–2.5_, and PM_1–2.5_; [20] lymphocytes and PNC [17] and PM_2.5_; [18] increased neutrophil counts with PNC; [18] and eosinophil counts with PM_2.5_ [17,18] and PNC [18]. Measurements of leukocyte, lymphocyte, neutrophil, and eosinophil counts may be useful in determining relationships between indoor air pollutant exposures and inflammation.

*Monocyte activation* plays an important role in inflammation. CD11b, CD31, CD62/CD62L, and CD49/CD49d are different types of expressions of adhesion markers found on monocytes. Two studies evaluated the different air exposures during active filtration and sham filtration [15,16], while one study monitored pollutants over time [17]. Three studies examined the association between these biomarkers and PM_2.5_ and PNC [15–17]. Two studies detected associations between CD11b with PM_2.5_ [16] and PNC [17]. An association with CD62L and active filtration was also detected, though biomarker concentrations were not analyzed against PM_2.5_ concentrations [15]. No association was reported with CD49/CD49d or CD31. More research is needed to determine if there may be an association between monocyte activation and indoor air exposures.

*Monocyte chemoattractant protein-1 (MCP-1)* regulates migration and infiltration of monocytes/macrophages [27] while *myeloperoxidase (MPO)* is an enzyme released by neutrophils during inflammation [28]. One study measured these two biomarkers alongside PM_2.5_ to compare true and sham air filtrations in dormitories of college students [14]. An association was detected between a decrease in MCP-1 and MPO during the true filtration scenario and an increase in MCP-1 with continuous exposure to PM_2.5_ [14].

*Urine leukotriene E4 (uLTE4)* is used to assess changes in cysteinyl-leukotriene levels [29]. One study measured uLTE4 to evaluate VOC indoor air exposures on airway inflammation by measuring urine and indoor VOCs 7 days pre- and post-move from an old to new hospital [30]. Although levels of uLTE4 significantly increased, no correlations were observed between VOCs and uLTE4 [30]. While uLTE4 may play a role in environmental exposures related to asthma [29,30], there is insufficient evidence to support its use in studies of indoor air exposures.

## Thrombosis and Coagulation

Three studies measured *von Willebrand Factor* (*vWF)* in office, dormitory, and home settings: [12,20,23] two compared different ventilation systems [12,23] while one monitored pollutants over time [20]. All three papers measured PM_2.5_, and two additionally measured O_3_ [12,23]. Other exposures measured included: NO_2_ and PNC [23], PM_10_, PM_10–2.5_, PM_1–2.5_, and PM_1_ [20]. All three papers showed significant associations: vWF was weakly associated with PM_1–2.5_, PM_2.5_, PM_10–2.5_, and PM_10_; [20] true filtration significantly lowered vWF by 26.9% when compared to sham filtration; [23] and removal of an electrostatic precipitator (ESP) was significantly associated with an increase in vWF [12]. This suggests PM_2.5_ can interfere with hemostasis by preventing the creation of the platelet plug. Of the hemostatic biomarkers reviewed, IAQ exhibited the strongest association with vWF.

Soluble adhesion molecule *P-selectin* (also known as sCD62P) binds vWF, acting as an anchor to the surface of endothelial cells for platelet adhesion [31]. Three studies studied the association of PM_2.5_ with P-selectin in office, dormitories, and homes and compared filtration systems [12,14,23]. O_3_ and PNC were also measured [12,23]. A 793 ppb/hr O_3_ exposure increase was associated with a 16.1% increase in P-selectin [12]. With PM_2.5_ exposure, no change in this biomarker was detected [14,23]. Two studies [12,23] also suggested O_3_ exposure may impact the binding of vWF to endothelial cells, but more research is needed on PM_2.5_ and its possible effect on P-selectin.

*Soluble CD40 ligand (sCD40L), plasminogen activator inhibitor-1 (PAI-1), tissue plasminogen activator (t-PA), and D-Dimer* were measured when comparing true and sham filtration systems in dormitories over a 2-day period [14]. Both sCD40L and *t*-PA significantly increased with an increase in PM_2.5_, while D-Dimer and PAI-1 showed no association [14]. Further research is needed to better understand the relationship between the fibrinolytic system and PM_2.5_.

## Oxidative Stress

8-hydroxy-2′-deoxyguanosine (8-OHdG) is a marker of oxidative stress that can be detected in blood or urine [24,32,33]. Eleven studies measured 8-OHdG; four compared functioning filtration system with a sham filtration system or control [12,13,19,33], four compared different populations based on occupation [25,32,34,35], one study monitored pollutants over time [24], one compared windows open, windows closed, and AC on conditions [21], and one report compared air exposures in different buildings [30]. Indoor air exposures included PM_1_ [33], PM_2.5_ [12,13,19,21,24,33,35], PM_10_ [21,25,33,34], polyaromatic hydrocarbons (PAHs) [32,33,35], VOCs [19,21,24,25,30], O_3_ [12], CO [21,24], CO_2_ [21,24], black carbon [13], and PNCs [35]. Seven studies detected association between 8-OHdG and the following air pollutants: PM_1_ [33], PM_2.5_ [19,21,33], VOCs [19,21,25], PAHs [32,33,35], UFPs [35], and CO_2_ [24]. 8-OHdG was frequently associated with changes in indoor air pollution, suggesting it may be of value for IAQ studies.

*Malondialdehyde (MDA)* is a product of lipid peroxidation that can be detected in blood or urine [26,35]. Six studies measured MDA: four in a home setting [23,33–35], one in an office and dormitory [12], and one in a hospital setting [30]. Two studies compared different participant occupations [34,35], two studies compared HEPA with sham filtration [12,23], one study compared air exposures in different buildings [30], and one study compared exposures before and after installation of a cooking emissions control device [33]. PM_1_ [33], PM_2.5_ [12,23,33,35], PM_10_ [33,34], O_3_ [12], PAHs [35], PNCs [23,35], and VOCs [30] were measured in these studies. A significant association was reported between MDA and the following indoor air exposures: PM_10_ [34] and the PAH benzo(a)pyrene (BaP) [33]. Additional oxidative stress biomarkers measured in one study included binucleated micronucleus (BNMN) frequency, comet tail length, comet tail DNA %, and superoxide dismutase (SOD) [34]. An association with PM was detected solely for comet tail length. However, there was a significant difference found in BNMNs and tail length when comparing kitchen workers and non-kitchen workers [34]. Both BNMNs and tail length were significantly higher in kitchen workers that were exposed to cooking oil fumes. While 8-OHdG and MDA appear to be valuable biomarkers to assess oxidative stress in indoor air exposures, more research is needed on other markers.

## Other Biomarkers

*Catecholamines (epinephrine and norepinephrine) and cortisol* were found to be associated with CO_2_ concentration in office space [24]. Biomarkers were not measured individually, so it is unclear if CO_2_ was associated with epinephrine, norepinephrine, or cortisol alone. This report suggests a relationship between urinary catecholamine and CO_2_ exposure, but more research is clearly needed on this topic.

*Clara cell pneumoproteins (CC16) and surfactant protein D (SPD)* are produced in the lungs and denote epithelial damage in the lower airways. Two studies evaluated their relationship with residential filtration, compared functioning filtration systems to sham filtration systems and measured PM_2.5_, and PNC of particles with diameters between 10 and 280 nm [15,16]. No association was detected between these biomarkers and filtration systems, PM_2.5_ exposure, or PNC exposure [15,16]. While SPD and CC16 are associated with chronic obstructive pulmonary disease [36,37], available data do not support their use in studies of indoor air exposures. *Angiotensin-converting enzyme and endothelin-1* were also measured when comparing true and sham filtration systems in dormitories over a 2-day period, but showed no association with PM_2.5_ [14].

*Glycosylated hemoglobin (HbA1c)*, was measured in urban homes of volunteers in Denmark. PM_2.5_ [15,17] and PNC [17] were monitored and an association with HbA1c was detected only for PNC. Thus, while recent studies reported an association between diabetes mellitus and air pollution, available data do not support the use of HbA1c in studies of indoor air exposures.

*Cyclic 3’: 5’ guanosine monophosphate (cGMP)* can increase when soluble guanylate cyclase is activated, which occurs with exposure to CO or NO [38,39]. One study examined differences in levels of chronic exposure to CO across four types of residential heating (piped natural gas, coal, electricity, and liquid propane gas) and its association with cGMP; [38] cGMP was higher in homes heated with liquid propane than in those heated with piped natural gas. However, CO exposures in the homes were too low to be the cause of this change, so it was hypothesized that NO may be a confounding factor [38]. NO can trigger the production of cGMP, but there is not enough research to determine if CO also triggers this production [39,40]. While cGMP may be a good indicator for NO exposure, more research is needed to determine if the biomarker is a good indicator of CO exposure.

## Organic Compounds

**Fig. 3.**
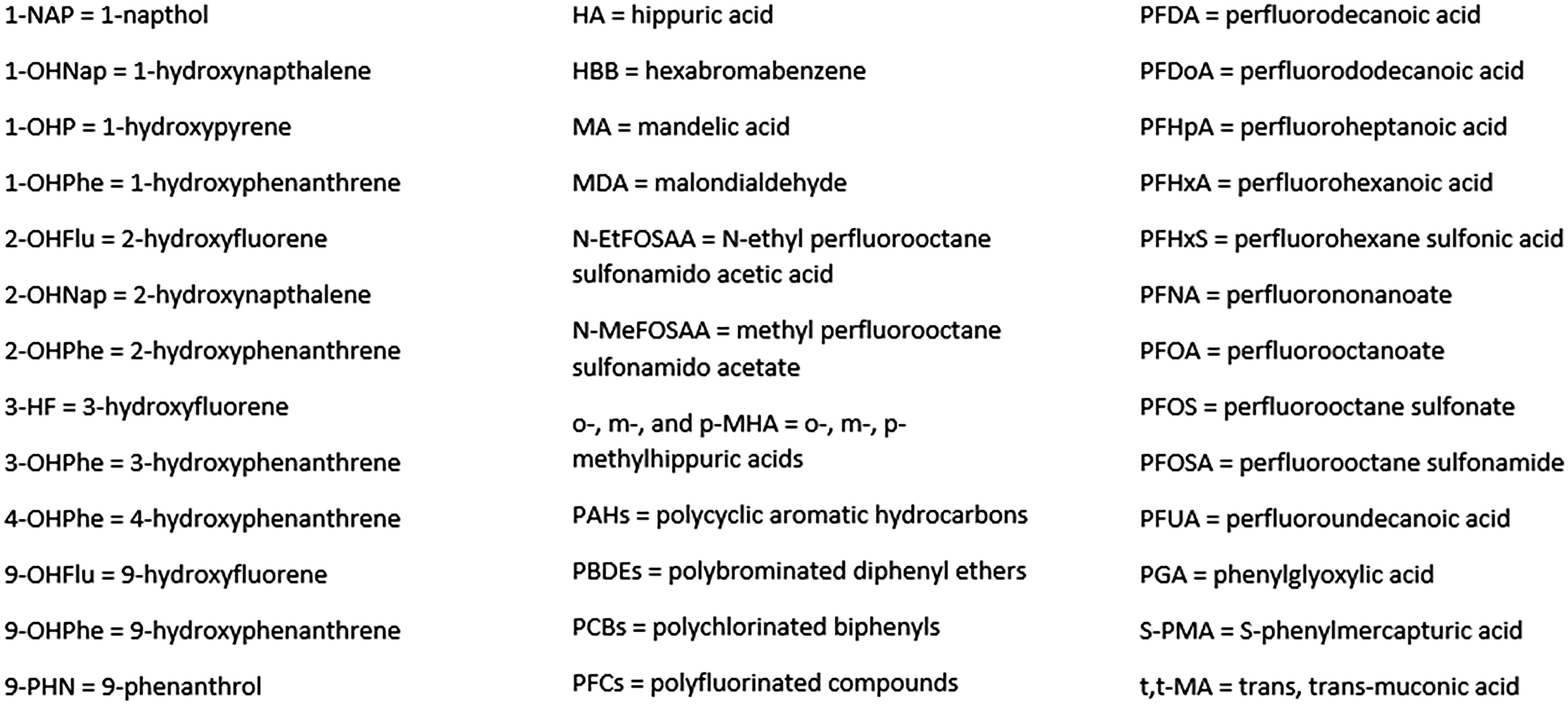
Glossary of organic compounds.

Indoor exposure to organic compounds (Fig. 3) can lead to measurable concentrations of these compounds or their metabolites in the blood or urine. Two studies measured office spaces’ PFCs and blood biomarkers PFNA and PFOS [41,42] (Table 1). Both studies compared air exposures in new buildings, partially new buildings, and old buildings while one study [41] additionally collected dust samples from participants’ offices, homes, and vehicles. Serum PFCs followed a consistent pattern with the FTOHs in the buildings’ air [42]. Serum PFOA was significantly associated with 8:2FTOH and 10:2FTOH [41] and positively associated with time spent in the office each week, suggesting PFOA bioaccumulation in participants [42]. Blood PFDA, PFOS, and PFHxS concentrations had no significant association with air PFCs [42].

Thirty-three PCB compounds were measured across three studies. One study evaluated the association between residential air PCBs and serum PCB compounds in high and low PCB areas [43], another study evaluated PCB exposure and blood between residents of PCB-contaminated and non-contaminated flats [44], and another study investigated the association between office air PCBs and office workers’ blood [45]. PCB 28 was the only measured compound that was reported to have statistical significance in all three studies.

Two studies compared household air samples to residents’ PBDE blood samples [46,47]. BDE-47 and BDE-99 showed significant associations with air PBDE [47]. Eight halogenated flame retardants were detected in participants’ serum, but none were associated with home PDBE exposures [46].

Thirteen urine PAH biomarkers were measured across seven papers [25,32,34,35,48–50]. Two studies [48,49] assessed PAH exposure and urinary PAH levels in kitchen and non-kitchen workers, while one study measured indoor PM_2.5_-bound PAH concentrations in dormitories, offices, and laboratories alongside urinary OH-PAHs [50]. The other five studies are described above [25,32,34,35,50]. Five papers showed significance between 1-OHP and indoor PAH exposures [32,35,48,49], and benzene, toluene, xylene in urban housemaids [25]. Three studies measured the remaining 12 PAH biomarkers [48–50]. 2-OHFlu, 9-OHFlu, 1-NAP, 9-PHN, and 3-HF showed significant associations with air PAHs [48,49] while 1-OHNap, 2-OHNap, 9-OHFlu, 4-OHPhe, and 9-OHPhe showed significant associations with exhaled FeNO [50]. 1-OHPhe, 2-OHPhe, and 3-OHPhe showed no associations with air exposures. The literature, alongside a 2004 review [51], suggests 1-OHP is a reliable biomarker when measuring indoor PAHs.

Two benzene biomarkers found in the literature were t,t-MA and S-PMA; the studies were described previously [25,30]. A significant decrease in t,t-MA was seen after moving from an old to new building [30], but no significant associations were found between t,t-MA and other exposures. Significantly higher levels of S-PMA were seen in city housemaids compared to drivers, traders, and rural housemaids [25]. S-PMA concentration may be a better indicator of benzene exposure, and is supported in previous literature [26,52].

Gas-phase benzene, toluene, ethylbenzene, styrene, o-, m-, and p-xylenes were measured in one study along with their counterpart urinary biomarkers [30]. Only o-, m-, and p-MHA levels significantly increased after the move from an old to new building, along with an increase in levels of TVOCs and all individual VOCs [30].

## Discussion

The World Health Organization (WHO) defines biomarkers as “any measurement reflecting an interaction between a biological system and a potential hazard, which may be chemical, physical, or biological” [53]. Biomarkers can serve as surrogate endpoints if they are associated with clinical outcomes [54]. The present review focused on studies of biomarkers indicative of changes in indoor air pollution exposure and of responses such as inflammation, oxidative stress, and coagulation. These biomarkers, therefore, constitute attractive intermediate endpoints for studies of IAQ. Herein, we summarize the current evidence pertaining to blood, urine, and saliva biomarkers used in IAQ research.

Indoor air exposures are a mixture of ambient air pollution brought indoors via ventilation and infiltration and indoor generated pollution emitted from combustion (i.e., candles, stove, fireplace), building materials and furnishings, and human behaviors such as smoking, cooking, and cleaning products [55–61]. Common indoor air pollutants include inorganic gases [e.g., carbon monoxide (CO), carbon dioxide (CO_2_)], reactive gases (e.g., O_3_, nitric oxides (NO_X_)], a wide range of VOCs and semi-volatile organic compounds (SVOCs), and particulate matter (PM), ranging from about 1 nm to 10 µm in diameter. Some compounds, such as polycyclic aromatic hydrocarbons (PAHs), perfluorinated compounds (PFCs), polychlorinated biphenyl (PCBs), and polybrominated diphenyl ethers (PBDE), are found in both the gas and particulate phases depending on partitioning behavior and emission source.

Poor air quality is associated with adverse clinical outcomes, which however take a long time to accrue and are thus challenging to use in translational research studies. Hence, the ability to rely on biomarkers as surrogate endpoints is critical to the conduct of observational studies as well as interventions. A previous review suggested that common mechanisms included inflammation and oxidative stress [26]. However, this study combined indoor and outdoor air pollution and its applicability to other settings or to indoor air pollution only is uncertain.

The present review extends prior knowledge by summarizing available data on the associations between biomarkers and IAQ. The mechanistic pathways associated with variations in IAQ include inflammation, coagulation, and oxidative stress. These pathways are known to be associated with chronic diseases, including cardiovascular diseases, respiratory diseases, and cancers supporting the biological plausibility of these associations.

### Limitations, Strengths, and Applications

Some limitations of the reviewed studies should be mentioned. Most studies were cross-sectional and almost half of the studies measured biomarkers at only one time point during the course of the study. Methods varied considerably across studies and hence direct comparison was challenging. Randomized intervention studies measuring paired groups of individuals are recommended for future IAQ biomarker studies to reduce confounding variables and improve quality research. Additionally, power was mentioned in only 3 of the 30 reviewed papers, therefore precluding its systematic assessment. Six biomarkers were measured in more than one type of specimen (blood, urine, or saliva), however, methods of measurements were not compared across specimen type. Thus, it is unclear if one specimen is more useful in measuring a particular biomarker than the other.

Our review has a number of important strengths. We conducted a comprehensive literature review using a rigorous methodology. Our review provides the most current review of the literature over the last decade and useful guidance for the selection of biomarkers in translational studies of IAQ.

### Conclusion

Herein, we summarize the current evidence on the biomarkers which most frequently responded to variations in IAQ. The biomarkers which exhibit the most consistent association with IAQ were high sensitivity CRP, vWF, 8-OHdG, and 1-hydroxypyrene (1-OHP. This summary provides a guide to select the biomarkers for translational studies evaluating the impact of indoor air pollutants on human health.

## Supplementary material

For supplementary material accompanying this paper visit https://doi.org/10.1017/cts.2020.532. click here to view supplementary material
