# Supplementary material for: Biomarkers and indoor air quality: A translational research review
Source: J Clin Transl Sci. 2020 Sep 4;5(1):e39. doi: 10.1017/cts.2020.532 (PMC8057458; doi:10.1017/cts.2020.532)
Supplement: Supplementary file 1 [file S2059866120005324sup001.docx]

**Appendix 1. Search Strategies**

Database(s): **Embase**1988 to 2019 Week 37**, Ovid MEDLINE(R) and Epub Ahead of Print, In-Process & Other Non-Indexed Citations**1996 to September 16, 2019**, EBM Reviews - Cochrane Central Register of Controlled Trials**August 2019**, EBM Reviews - Cochrane Database of Systematic Reviews**2005 to September 11, 2019

| **#** | **Searches** |
| --- | --- |
| 1 | Air Pollutants/ or air pollutant/ or air pollution/ or ((ambient or air) adj7 (pollut* or quality or particulat* or particle* or "carbon dioxide" or "carbon monoxide" or "nitrogen oxide" or "nitrogen dioxide pr hydrocarbon*" or PAH* or aerosol* or phthalate* or polyfluoroalkyl or PFAS or bisphenol or "black carbon")).ti,ab,hw,kw,kf. |
| 2 | Occupational Exposure/ or ((occupation* or worker*) adj3 expos*).ti. |
| 3 | 1 and 2 |
| 4 | Air Pollutants, Occupational/ |
| 5 | 3 or 4 |
| 6 | (household* or home or homes or kitchen* or cook* or office* or building* or indoor*).ti,ab,hw,kw,kf. |
| 7 | 5 and 6 |
| 8 | ((household* or home or homes or kitchen* or cook* or office* or building* or indoor*) adj3 (ambient or air) adj3 (pollut* or quality or particulat* or particle* or "carbon dioxide" or "carbon monoxide" or "nitrogen oxide" or "nitrogen dioxide pr hydrocarbon*" or PAH* or aerosol* or phthalate* or polyfluoroalkyl or PFAS or bisphenol or "black carbon")).ti,ab,hw,kw,kf. |
| 9 | ((household* or home or homes or kitchen* or cook* or office* or building* or indoor*) and (ambient or air) and (pollut* or quality or particulat* or particle* or "carbon dioxide" or "carbon monoxide" or "nitrogen oxide" or "nitrogen dioxide pr hydrocarbon*" or PAH* or aerosol* or phthalate* or polyfluoroalkyl or PFAS or bisphenol or "black carbon")).ti. |
| 10 | *ambient air/ or *Air Pollution, Indoor/ |
| 11 | or/8-10 |
| 12 | 7 or 11 |
| 13 | Blood Coagulation Factors/ |
| 14 | biomarkers/ |
| 15 | Urine/an [Analysis] |
| 16 | blood/ |
| 17 | exp plasma/ |
| 18 | serum/ |
| 19 | Saliva/ |
| 20 | exp Inflammation/ |
| 21 | ((inflammation or inflammatory) adj3 (marker* or biomarker*)).ti,ab,hw,kw,kf. |
| 22 | "Polycyclic Aromatic Hydrocarbons"/ur |
| 23 | "8-OHdG".ti,ab,hw,kw,kf. |
| 24 | ("urinary 8-hydroxydeoxyguanosine" or "8-hydroxy-2-deoxyguanosine").ti,ab,hw,kw,kf. |
| 25 | (urine or blood or plasma* or serum* or saliva*).ti. |
| 26 | ("Interleukin-8" or "IL-8" or "Interleukin-6" or "IL-6" or "von Willebrand factor" or "PAH metabolite*" or "sCD62P" or Cotinine or Carboxyhemoglobin or COHb or "ET 1-21" or "P-selectin" or "Leukotriene B4" or "C Reactive Protein*" or Fibrinogen or Malondialdehyde).ti,ab,hw,kw,kf. |
| 27 | or/13-26 |
| 28 | 12 and 27 |
| 29 | limit 28 to english language [Limit not valid in CDSR; records were retained] |
| 30 | limit 29 to yr="2000 -Current" |
| 31 | 30 not ((exp animals/ or exp nonhuman/) not exp humans/) |
| 32 | limit 31 to (conference abstract or editorial or erratum or note or addresses or autobiography or bibliography or biography or blogs or comment or dictionary or directory or interactive tutorial or interview or lectures or legal cases or legislation or news or newspaper article or overall or patient education handout or periodical index or portraits or published erratum or video-audio media or webcasts) [Limit not valid in Embase,Ovid MEDLINE(R),Ovid MEDLINE(R) In-Process,Ovid MEDLINE(R) Publisher,CCTR,CDSR; records were retained] |
| 33 | 31 not 32 |
| 34 | remove duplicates from 33 |

**SCOPUS**

| 1 | TITLE-ABS-KEY ( ( household* OR home OR homes OR kitchen* OR cook* OR office* OR building* OR indoor* ) W/3 ( ambient OR air ) W/3 ( pollut* OR quality OR particulat* OR particle* OR "carbon dioxide" OR "carbon monoxide" OR "nitrogen oxide" OR "nitrogen dioxide pr hydrocarbon*" OR pah* OR aerosol* OR phthalate* OR polyfluoroalkyl OR pfas OR bisphenol OR "black carbon" ) ) |
| --- | --- |
| 2 | TITLE ( ( household* OR home OR homes OR kitchen* OR cook* OR office* OR building* OR indoor* ) AND ( ambient OR air ) AND ( pollut* OR quality OR particulat* OR particle* OR "carbon dioxide" OR "carbon monoxide" OR "nitrogen oxide" OR "nitrogen dioxide pr hydrocarbon*" OR pah* OR aerosol* OR phthalate* OR polyfluoroalkyl OR pfas OR bisphenol OR "black carbon" ) ) |
| 3 | TITLE-ABS-KEY ( ( indoor* W/1 air ) ) |
| 4 | #1 or #2 or #3 |
| 5 | TITLE ( ( blood OR biomarker* OR urine OR plasma OR serum OR saliva OR inflammat* OR "urinary 8-hydroxydeoxyguanosine" OR "8-hydroxy-2-deoxyguanosine" OR "8-OHdG" OR "polycyclic aromatic hydrocarbon*" ) ) |
| 6 | TITLE ( "Interleukin-8" OR "IL-8" OR "Interleukin-6" OR "IL-6" OR "von Willebrand factor" OR "PAH metabolite*" OR "sCD62P" OR cotinine OR carboxyhemoglobin OR cohb OR "ET 1-21" OR "P-selectin" OR "Leukotriene B4" OR "C Reactive Protein*" OR fibrinogen OR malondialdehyde ) |
| 7 | #5 or #6 |
| 8 | #4 and #7 |
| 9 | INDEX(embase) OR INDEX(medline) OR PMID(0* OR 1* OR 2* OR 3* OR 4* OR 5* OR 6* OR 7* OR 8* OR 9*) |
| 10 | #8 and not #9 |
| 11 | DOCTYPE(ed) OR DOCTYPE(bk) OR DOCTYPE(er) OR DOCTYPE(no) OR DOCTYPE(sh) OR DOCTYPE(ch) |
| 12 | #10 and not #11 |
| 13 | LANGUAGE(english) |
| 14 | #12 and #13 |
| 15 | PUBYEAR aft 1999 |
| 16 | #14 and #15 |
